# Dietary Intake of Fatty Acids and Serum C-reactive Protein in Japanese

**DOI:** 10.2188/jea.17.86

**Published:** 2007-06-02

**Authors:** Satoko Yoneyama, Katsuyuki Miura, Satoshi Sasaki, Katsushi Yoshita, Yuko Morikawa, Masao Ishizaki, Teruhiko Kido, Yuchi Naruse, Hideaki Nakagawa

**Affiliations:** 1 Department of Epidemiology and Public Health, Kanazawa Medical University.; 2 Nutritional Epidemiology Program, National Institute of Health and Nutrition.; 3 Department of Social and Environmental Medicine, Kanazawa Medical University.; 4 Department of Community Health Nursing, Kanazawa University School of Health Science.; 5 Department of Community Health and Gerontological Nursing, Toyama University.

**Keywords:** C-Reactive Protein, Fatty Acids, Diet, Epidemiology

## Abstract

**BACKGROUND:**

Inflammation has been established as a risk factor for cardiovascular diseases. The relationships of polyunsaturated fatty acids (PUFAs) and monounsaturated fatty acids to inflammation are still controversial in Western populations. The relationships are not clear in Japanese whose intake of seafood-based long-chain n-3 PUFAs is high.

**METHODS:**

We conducted a cross-sectional epidemiologic study in the Japanese population (1,556 men and 1,461 women aged 35-60). Serum high sensitivity C-reactive protein (CRP) was measured, and intakes of 7 specific fatty acids (% of energy) were evaluated by a validated self-administered dietary history questionnaire.

**RESULTS:**

CRP was significantly and inversely related to the intakes of oleic acid (p=0.008) and *α* -linolenic acid (p=0.026) in women after adjustment for confounding factors. A multiple regression analysis showed that, especially in the middle tertile of long-chain n-3 PUFAs (eicosapentaenoic acid and docosahexaenoic acid) intake, CRP was inversely related to the intake of oleic acid and linoleic acid in both sexes and to the intake of *α* -linolenic acid in women.

**CONCLUSION:**

Intakes of oleic acid, linoleic acid, and *α* -linolenic acid would reduce serum CRP, especially when the intake of long-chain n-3 PUFAs is at a moderate level in Japanese.

Inflammation plays an important role in all stages of atherosclerosis, and C-reactive protein (CRP), a marker of systemic inflammation, has been shown to predict cardiovascular events.^1^^-^^4^ It has been reported that higher CRP is related to older age, obesity, and smoking,^5^^,^^6^ but what dietary factors increase CRP are still not clear. Because polyunsaturated fatty acids (PUFAs) and monounsaturated fatty acids (MUFAs) are suggested to retard inflammation, several cross-sectional and intervention studies have been reported from Western countries. Some intervention studies reported that supplementation of *α* -linolenic acid (ALA), a precursor of long-chain n-3 PUFAs, reduced CRP,^7^^-^^10^ but effects of n-6 PUFAs (such as linoleic acid), long-chain n-3 PUFAs (such as eicosapentaenoic acid [EPA] and docosahexaenoic acid [DHA]), and MUFAs (such as oleic acid) have been controversial.^11^

On the other hand, PUFA intake may reduce cardiovascular disease risk, but it has been suggested that n-6 PUFAs may compete with n-3 PUFA metabolism and attenuate benefits. Additionally, seafood-based long-chain n-3 PUFAs (EPA and DHA) may modify the effects of plant-based, intermediate-chain n-3 PUFAs (ALA). A recent study examined the interactions of these PUFAs in relation to coronary heart disease risk.^12^ MUFAs (n-9 fatty acids) may also compete with PUFAs for common metabolic enzymes. Controversies on the relationship of MUFAs and PUFAs to CRP may have arisen because most previous studies were conducted in Western countries where long-chain n-3 PUFA intake is relatively low.

The Japanese diet is rich in seafood, and is therefore correspondingly rich in seafood-based long-chain n-3 PUFAs. For example, Japanese consume 5-8 times more EPA and DHA (0.8-0.9 g/day) than people in the United States (0.1-0.2 g/day).^13^^,^^14^ This large-scale cross-sectional epidemiologic study in Japanese is designed to clarify the relationships between the intake of various fatty acids and serum CRP in Japanese, whose diet is rich in EPA and DHA, and to investigate whether intake of EPA and DHA influences the relationship between CRP and intake of various other fatty acids.

## METHODS

Study subjects were male and female workers aged 35-60 years employed at a zipper and aluminum sash-producing factory in Japan. In 2002-2003, they underwent health examinations and provided blood sample, and we asked additional questions about medications, physical activity, and smoking status (current, stopped, never). The body mass index (BMI) was calculated as weight (kg)/height^2^ (m^2^). Physical activity was assessed in hours per week spent on common leisure time physical activities expressed as metabolic equivalent hours per week (MET-h/wk).

Dietary habits during the previous month were assessed using a self-administered diet history questionnaire (DHQ),^15^^,^^16^ which was completed by each subject at home and was checked by dietitians. The DHQ is a 16-page structured questionnaire that consists of the following 7 sections: general dietary behaviors; major cooking methods; consumption frequency and amount of 6 alcoholic beverages; semi-quantitative frequency of intake of 121 selected food and nonalcoholic beverage items; dietary supplements; consumption frequency and amount of 19 staple foods (rice, bread, noodles, and other wheat foods) and “miso” (fermented soybean paste) soup; and open-ended items for foods consumed regularly (≥ once/wk) but not appearing in the DHQ. The food and beverage items and portion sizes in the DHQ were derived primarily from data in the National Nutrition Survey of Japan and several recipe books for Japanese dishes.^15^ Measures of dietary intake for 148 food and beverage items, energy, energy-providing nutrients including each fatty acid, and non energy-providing nutrients such as calcium, iron, sodium, vitamin A, vitamin C, and dietary fiber, were calculated using an ad hoc computer algorithm developed for the DHQ, which was based on the Standard Tables of Food Composition in Japan.^17^ Information on dietary supplements and data from the open-ended questionnaire items were not used in the calculation of dietary intake. Detailed descriptions of the methods used for calculating dietary intake and the validity of the DHQ have been published elsewhere.^15^^,^^16^ In this study, we used energy density value for nutrient intakes (% of energy [%E] for energy-providing nutrients and weight per 1000kcal for non energy-providing nutrients).

Serum high-sensitivity CRP was measured via a high-sensitivity latex-enhanced immunonephelometric assay on Behring Nephelometer Analyzer II (Dade Behring, Marburg, Germany).

Among 4,984 workers aged 35-60 years, nutritional data by DHQ were obtained in 4,639 participants (93%) and serum CRP was measured in 3,388 participants (68%). Complete data for analysis were obtained in 3,057 participants (61%). Forty participants with a CRP concentration 10+mg/L were excluded due to the possibility of other source of inflammation. Finally, the study analyses were performed in 3,017 participants, 1,556 men and 1,461 women. We used log-transformed values for CRP and some of the dietary variables to account for their skewed distribution. We selected 5 fatty acids, where intake was over 0.6%E, and EPA and DHA, which are important for our hypothesis, for analysis. The intake of each fatty acid was divided into quintiles, and geometric means of CRP were compared among the quintiles, using analysis of covariance adjusted for age, BMI, alcohol consumption, smoking status, and leisure time physical activity, where the results of multiple comparison tests versus the lowest quintile were also shown. Smoking status were adjusted using dummy variables. Multiple liner regression analysis was used to assess the relation between CRP and major MUFA and PUFA intake in each of three groups with lower, middle, and higher intakes (tertiles) of long-chain n-3 PUFAs (EPA+DHA). These analyses were adjusted for age, BMI, alcohol consumption, smoking status, leisure time physical activity, and intake of saturated fatty acids (SFAs). All analyses were performed in men and in women, separately. Statistical analysis was performed using the Statistical Analysis System^®^ (SAS Institute Inc., Cary, North Carolina, USA).

## RESULTS

Basic characteristics and dietary intake of nutrients are shown in Table 1. Mean age was 47.0 years in both sexes. Geometric mean of serum CRP was significantly higher in men (0.43mg/L) than in women (0.27mg/L) (p<0.001). Intake of protein, calcium, iron, sodium, vitamin A, vitamin C, and cholesterol were significantly higher in women than in men (p<0.001). In contrast, carbohydrate (p=0.02) and alcohol (p<0.001) were significantly higher in men than in women. Serum CRP was positively related to age in women (p<0.001), BMI in both sexes (p<0.001), and smoking in men (p<0.001) (data not shown).

**Table 1.** Basic characteristics and major nutrients intake.

	Men (n=1556)	Women (n=1460)	p^‡^	All (n=3016)
		
	Mean (95% CI)	Mean (95% CI)		Mean (95% CI)
Age (year)	47.0 (46.7 - 47.3)	47.0 (46.7 - 47.4)	0.870	47.0 (46.8 - 47.2)
Height (cm)	168.8 (168.5 - 169.1)	155.5 (155.2 - 155.8)	<0.001	162.3 (162.0 - 162.7)
Weight (kg)	67.0 (66.5 - 67.5)	54.9 (54.4 - 55.3)	<0.001	61.1 (60.7 - 61.5)
Body mass index (kg/m^2^)	23.5 (23.3 - 23.6)	22.7 (22.5 - 22.9)	<0.001	23.1 (23.0 - 23.2)
Leisure time physical activity (MET-h/week)*	1.0 (0.9 - 1.1)	0.8 (0.7 - 0.8)	<0.001	0.9 (0.8 - 1.0)
Current smoking (%)^†^	56.1	3.9	<0.001	30.8
Systolic blood pressure (mmHg)	124.2 (123.5 - 124.9)	119.4 (118.6 - 120.2)	<0.001	121.8 (121.3 - 122.4)
Diastolic blood pressure (mmHg)	76.7 (76.1 - 77.2)	72.9 (72.4 - 73.5)	<0.001	74.9 (74.5 - 75.3)
Serum total cholesterol (mg/dL)	205.9 (204.3 - 207.6)	207.3 (205.6 - 209.0)	0.248	206.6 (205.4 - 207.8)
Serum HDL-cholesterol (mg/dL)	59.2 (58.4 - 60.0)	68.1 (67.4 - 68.9)	0.829	63.5 (62.9 - 64.1)
Serum CRP (mg/L)*	0.43 (0.41 - 0.45)	0.27 (0.25 - 0.28)	<0.001	0.34 (0.33 - 0.36)

Total energy (kcal)	2161 (2128 - 2194)	1846 (1814 - 1879)	<0.001	2008 (1984 - 2032)
Protein (%E)	15.7 (15.5 - 15.8)	17.9 (17.8 - 18.1)	<0.001	16.8 (16.6 - 16.9)
Carbohydrate (%E)	61.0 (60.5 - 61.4)	60.3 (59.9 - 60.7)	0.024	60.6 (60.3 - 60.9)
Calcium (mg/1000kcal)*	169.0 (166.9 - 173.2)	235.7 (232.9 - 240.6)	<0.001	198.6 (196.9 - 202.3)
Iron (mg/1000kcal)	2.84 (2.81 - 2.88)	3.54 (3.50 - 3.58)	<0.001	3.18 (3.15 - 3.21)
Sodium (mg/1000kcal)	1782 (1756 - 1808)	2118 (2089 - 2146)	<0.001	1944 (1924 - 1964)
Vitamin A (IU/1000kcal)*	2096 (2006 - 2189)	2595 (2509 - 2684)	<0.001	2325 (2260 - 2390)
Vitamin C (mg/1000kcal)*	29.9 (29.0 - 30.9)	45.8 (44.6 - 47.0)	<0.001	36.8 (36.0 - 37.6)
Alcohol (%E)*	4.19 (3.91 - 4.48)	0.50 (0.45 - 0.55)	<0.001	1.84 (1.73 - 1.96)
Dietary fiber (mg/1000kcal)	5.08 (5.01 - 5.16)	6.46 (6.38 - 6.55)	<0.001	5.75 (5.69 - 5.81)
Dietary cholesterol (mg/1000kcal)	94.0 (91.9 - 96.2)	118.9 (116.5 - 121.3)	<0.001	106.1 (104.4 - 107.8)

* : Geometric means

† : Percentages of current smokers

‡ : Statistical difference between men and women by Student’s t-test, by Wilcoxon test (for variables with*), or by chi-square test (for smoking status)

CI : confidence interval

CRP : C-reactive protein

%E : percent of energy

Mean values for fatty acid intake are shown in Table 2. Total fat intake was 20.8 %E in men and 27.0 %E in women. The ratio of n-6/n-3 in men and women were 4.44 and 4.35, respectively. Among specific fatty acids, higher intakes were observed for oleic acid (6.1 %E in men and 7.8 %E in women) and for linoleic acid (4.6 %E in men and 5.8 %E in women). Mean absolute amounts of fatty acids intake in men and in women were 1.81 g and 1.98 g for ALA, 0.34 g and 0.38 g for EPA, and 0.46 g and 0.51 g for DHA, respectively.

**Table 2.** Intake of fatty acids.

	Men	Women	p^†^	All
		
	Mean (95% CI)	Mean (95% CI)		Mean (95% CI)
Total fat (%E)	20.8 (20.5 - 21.1)	27.0 (26.7 - 27.3)	<0.001	23.8 (23.5 - 24.1)
Saturated fatty acids (%E)	5.08 (4.99 - 5.17)	6.77 (6.67 - 6.87)	<0.001	5.90 (5.82 - 5.97)
Monounsaturated fatty acids (%E)	7.15 (7.02 - 7.28)	9.16 (9.03 - 9.30)	<0.001	8.13 (8.03 - 8.22)
Polyunsaturated fatty acids (%E)	5.44 (5.35 - 5.52)	6.78 (6.69 - 6.87)	<0.001	6.09 (6.02 - 6.15)
n-3 PUFAs (%E)	1.14 (1.11 - 1.16)	1.44 (1.42 - 1.47)	<0.001	1.28 (1.27 - 1.30)
n-6 PUFAs (%E)	4.73 (4.66 - 4.80)	5.97 (5.89 - 6.05)	<0.001	5.33 (5.27 - 5.39)
(n-6)/(n-3) ratio	4.44 (4.38 - 4.50)	4.35 (4.30 - 4.40)	0.022	4.40 (4.36 - 4.43)

Palmitic acid (C16:0) (%E)	3.31 (3.26 - 3.37)	4.33 (4.27 - 4.38)	<0.001	3.80 (3.76 - 3.85)
Stearic acid (C18:0) (%E)	1.25 (1.22 - 1.27)	1.69 (1.66 - 1.72)	<0.001	1.46 (1.44 - 1.48)
Oleic acid (C18:1) (%E)	6.11 (5.99 - 6.22)	7.82 (7.70 - 7.95)	<0.001	6.94 (6.85 - 7.03)
Linoleic acid (C18:2 n-6) (%E)	4.59 (4.52 - 4.66)	5.75 (5.67 - 5.82)	<0.001	5.15 (5.09 - 5.21)
*α* -Linolenic acid (C18:3 n-3) (%E)	0.77 (0.75 - 0.78)	0.97 (0.96 - 0.99)	<0.001	0.87 (0.86 - 0.88)
Eicosapentaenoic acid; EPA (C20:5 n-3) (%E)*	0.137 (0.131 - 0.142)	0.177 (0.171 - 0.184)	<0.001	0.156 (0.152 - 0.160)
Docosahexaenoic acid; DHA (C22:6 n-3) (%E)*	0.185 (0.179 - 0.191)	0.238 (0.231 - 0.245)	<0.001	0.210 (0.205 - 0.215)

* : Geometric means

† : Statistical difference between men and women by Student’s t-test or by Wilcoxon test (for variables with*)

CI : confidence interval

%E : percent of energy

PUFA : polyunsaturated fatty acids

Multivariate-adjusted geometric means of serum CRP by quintiles of each fatty acid intake are shown in Table 3. In women, there were significant decreasing tendencies in serum CRP as the quintiles of intakes of total fat, SFAs, MUFAs, and PUFAs increased. For specific fatty acids, significant inverse relationships were observed for oleic acid (p=0.008), and ALA (p=0.026) in women (Table 3, lower panel). Although these relationships were not statistically significant, similar tendencies were observed also in men (Table 3, upper panel). Intakes of EPA and DHA were not significantly related to CRP in both sexes.

**Table 3.** Multivariate-adjusted mean values of serum CRP by quintiles (Q1-Q5) of each fatty acid intake.

	Q1 (lowest)	Q2	Q3	Q4	Q5 (highest)	p for trend
	Men					
Total fat	0.48	0.42	0.42	0.43	0.40*	0.332
Saturated fatty acids	0.48	0.43	0.39**	0.43	0.43	0.131
Monounsaturated fatty acids	0.48	0.42	0.41	0.44	0.42	0.387
Polyunsaturated fatty acids	0.47	0.43	0.43	0.40	0.42	0.493
n-3 PUFAs	0.42	0.47	0.40	0.43	0.43	0.409
n-6 PUFAs	0.46	0.43	0.45	0.41	0.41	0.514
(n-6)/(n-3) ratio	0.49	0.45	0.43	0.38**	0.42	0.038

Palmitic acid (C16:0)	0.48	0.43	0.42	0.42	0.41	0.448
Stearic acid (C18:0)	0.49	0.41*	0.43	0.42	0.41*	0.210
Oleic acid (C18:1)	0.47	0.44	0.39*	0.46	0.42	0.138
Linoleic acid (C18:2 n-6)	0.48	0.41	0.43	0.42	0.42	0.358
*α* -Linolenic acid (C18:3 n-3)	0.48	0.42	0.44	0.40*	0.42	0.314
Eicosapentaenoic acid (EPA) (C20:5 n-3)	0.42	0.44	0.45	0.39	0.47	0.136
Docosahexaenoic acid (DHA) (C22:6 n-3)	0.42	0.44	0.45	0.40	0.45	0.508

	Women					
Total fat	0.31	0.26*	0.23***	0.30	0.24***	<0.001
Saturated fatty acids	0.32	0.25**	0.24**	0.26*	0.28	0.009
Monounsaturated fatty acids	0.31	0.27*	0.24^**^	0.27	0.24**	0.011
Polyunsaturated fatty acids	0.31	0.26	0.25*	0.27	0.24**	0.034
n-3 PUFAs	0.32	0.26**	0.26*	0.25**	0.26*	0.020
n-6 PUFAs	0.31	0.26*	0.27	0.26*	0.24**	0.067
(n-6)/(n-3) ratio	0.29	0.28	0.25	0.27	0.25	0.447

Palmitic acid (C16:0)	0.31	0.26*	0.25*	0.25*	0.27	0.095
Stearic acid (C18:0)	0.30	0.27	0.25*	0.26	0.26	0.170
Oleic acid (C18:1)	0.31	0.26*	0.24**	0.29	0.24**	0.008
Linoleic acid (C18:2 n-6)	0.31	0.27	0.26	0.26	0.24**	0.060
*α* -Linolenic acid (C18:3 n-3)	0.31	0.26*	0.26*	0.24**	0.26*	0.026
Eicosapentaenoic acid (EPA) (C20:5 n-3)	0.30	0.25*	0.26	0.27	0.25*	0.224
Docosahexaenoic acid (DHA) (C22:6 n-3)	0.30	0.27	0.26	0.26	0.25	0.324

Values are geometric means of CRP (mg/L) by quintiles of each fatty acids intake (% of energy), adjusted for age, body mass index, alcohol consumption, smoking status and leisure time physical activity.

CRP : C-reactive protein

PUFA : polyunsaturated fatty acids

* : p<0.05, ** : p<0.01, *** : p<0.001 versus the lowest quintile (Q1)

Because we hypothesized that the intake of long-chain n-3 PUFAs may interact the relationships of CRP to other PUFAs and MUFAs, we assessed the relationships of three fatty acids (oleic acid, linoleic acid, and ALA) to serum CRP in each tertile of long-chain n-3 PUFAs (EPA+DHA) intake. Results are shown in Table 4 for men and in Table 5 for women. In men, oleic acid and linoleic acid were significantly and inversely related to serum CRP only in the middle tertile of EPA+DHA intake, in the SFA-adjusted model (model 2) (Table 4). In women, the intakes of oleic acid, linoleic acid, and ALA were significantly and inversely related to serum CRP in the lower and the middle tertiles of EPA+DHA intake (Table 5).

**Table 4.** Multiple linear regression analysis on serum CRP in relation to each of 3 fatty acids in each tertile of EPA+DHA intake (men).

	Lower EPA+DHA intake (-0.20%E) (n=502)			Middle EPA+DHA intake (0.21-0.38%E) (n=524)			Higher EPA+DHA intake (0.39+%E) (n=530)		
		
	Coefficient	t-value	p	Coefficient	t-value	p	Coefficient	t-value	p
Oleic acid (C18:1) (%E)									
Model 1	-0.007	-0.4	0.730	-0.038	-1.6	0.102	-0.006	-0.3	0.770
Model 2	-0.009	-0.3	0.734	-0.105	-2.6	0.009	0.020	0.4	0.699

Linoleic acid (C18:2 n-6) (%E)									
Model 1	-0.006	-0.2	0.839	-0.036	-1.0	0.321	-0.017	-0.5	0.599
Model 2	0.002	0.1	0.946	-0.109	-2.3	0.021	0.016	0.3	0.762

*α* -Linolenic acid (C18:3 n-3) (%E)									
Model 1	-0.025	-0.2	0.861	-0.144	-0.8	0.401	-0.032	-0.2	0.841
Model 2	0.008	0.1	0.948	-0.401	-1.9	0.059	0.103	0.4	0.667

CRP : C-reactive protein

EPA : eicosapentaenoic acid

DHA : docosahexaenoic acid

%E : percent of energy

Model 1 is adjusted for age, body mass index, alcohol consumption, smoking status and leisure time physical activity.

Model 2 is adjusted for variables in Model 1 plus saturated fatty acids.

**Table 5.** Multiple linear regression analysis on serum CRP in relation to each of 3 fatty acids in each tertile of EPA+DHA intake (women).

	Lower EPA+DHA intake (-0.29%E) (n=504)			Middle EPA+DHA intake (0.30-0.51%E) (n=451)			Higher EPA+DHA intake (0.52+%E) (n=505)		
		
	Coefficient	t-value	p	Coefficient	t-value	p	Coefficient	t-value	p
Oleic acid (C18:1) (%E)									
Model 1	-0.0410	-2.3	0.025	-0.0425	-2.0	0.052	-0.0031	-0.2	0.881
Model 2	-0.0478	-1.9	0.065	-0.0688	-2.2	0.028	0.0358	1.2	0.236

Linoleic acid (C18:2 n-6) (%E)									
Model 1	-0.0667	-2.5	0.012	-0.0892	-2.6	0.009	0.0220	0.7	0.486
Model 2	-0.0643	-2.2	0.032	-0.0978	-2.6	0.009	0.0545	1.5	0.129

*α* -Linolenic acid (C18:3 n-3) (%E)									
Model 1	-0.2726	-2.3	0.023	-0.3712	-2.5	0.015	0.1687	1.1	0.279
Model 2	-0.2469	-2.0	0.052	-0.3751	-2.4	0.018	0.2851	1.7	0.089

CRP : C-reactive protein

EPA : eicosapentaenoic acid

DHA : docosahexaenoic acid

%E : percent of energy

Model 1 is adjusted for age, body mass index, alcohol consumption, smoking status and leisure time physical activity.

Model 2 is adjusted for variables in Model 1 plus saturated fatty acids.

## DISCUSSION

In this study in a large Japanese population, whose seafood intake is higher compared to that in Western populations, significant and inverse relationships between serum CRP and intakes of total fat, SFAs, MUFAs, PUFAs, and some specific fatty acids were observed especially in women. Also, the significant inverse relationships of serum CRP were observed to oleic acid and linoleic acid in Japanese men with a moderate intake of long-chain n-3 PUFAs (EPA+DHA) and to oleic acid, linoleic acid, and ALA in Japanese women with a lower or moderate intake of long-chain n-3 PUFAs.

Because PUFAs and MUFAs are suggested to retard inflammation, several cross-sectional and intervention studies have been reported from Western countries. Especially for ALA (plant-based, intermediate-chain n-3 PUFA), there are randomized trials showing its anti-inflammatory effects.^7^^-^^10^ Although the amount of ALA supplementation in these trials was much higher than the usual dietary ALA intake, our results in Japanese subjects would support their findings. For seafood-based long-chain n-3 PUFAs (EPA and DHA), there have been several studies investigating the relationship to CRP. A cross-sectional study from the Nurses’ Health Study found that CRP was 32% lower in the highest quintile of EPA+DHA intake compared with the lowest quintile.^18^ However, intervention trials have not yet confirmed that EPA+DHA supplementation decreases CRP.^19^^-^^21^ Our study in Japanese subjects also did not show significant relationship of EPA and DHA to CRP.

The so-called Mediterranean-style diet has been reported to reduce cardiovascular risk, and a recent randomized trial reported on its anti-inflammatory effect.^22^ This diet is known to be rich in oleic acid, fiber, and antioxidants so that these nutrients may be beneficial in reducing inflammation. Because our results showed a relatively stronger relationship of oleic acid to CRP compared with other fatty acids, further investigation focusing on oleic acid intake is needed. For linoleic acid (plant-based n-6 PUFA), several randomized trials compared anti-inflammatory effect with ALA (n-3 PUFA),^7^^-^^9^ and a lower n-6/n-3 ratio has been recommended. However, it has also been shown that a linoleic acid rich diet reduced inflammation.^7^ Because our study showed a relatively strong inverse relationship of linoleic acid to CRP especially in women, the substitution of linoleic acid for SFAs may be recommended.

One of the most important purposes of this study was to investigate whether there is an interaction of long-chain n-3 PUFAs intake for the relationships between other fatty acids and CRP. Previous epidemiologic findings on ALA, linoleic acid, and oleic acid in Western countries may be affected by lower intake of EPA and DHA. Japanese have been reported to consume 5-8 times more long-chain n-3 PUFAs. In the United States, intake of n-3 PUFAs was 1.6 g/day (0.7 %E), of which 1.4g was ALA and 0.1-0.2 g was EPA+DHA. In Japan, intake of n-3 PUFAs was 2.8 g/day (2.3 %E), of which 1.9 g was ALA and 0.8-0.9 g was EPA+DHA.^13^^,^^14^ The INTERMAP study showed that EPA+DHA intake in Japan was 1.2 g/day in men and 0.9 g/day in women, whereas that in the USA was 0.2 g/day in both men and women.^23^ In the present study, the relationships of oleic acid, linoleic acid, and ALA to CRP were especially strong when EPA+DHA intake was the average for this population. However, it should be noted that this level is much higher than that in Western countries. If people in Western countries were to consume more EPA and DHA, the anti-inflammatory effects of oleic acid, linoleic acid, and ALA may be stronger.

The mechanisms underlying the relationship of these fatty acids to inflammation and of the interaction with EPA+DHA are yet to be fully elucidated. After ingestion, three types of 18-carbon fatty acids, i.e. oleic acid (18:1 n-9), linoleic acid (18:2 n-6), and ALA (18:3 n-3), are desaturated and elongated to 20-carbon fatty acids by common metabolic enzymes (*δ* 5-desaturase and *δ* 6-desaturase). Oleic acid is converted to eicosatrienoic acid (20:3 n-9); linoleic acid is converted to arachidonic acid (20:4 n-6); and ALA is converted to EPA (20:5 n-3).^24^^-^^26^ The pro-inflammatory eicosanoids prostaglandin E2 and leukotriene B4 are derived from arachidonic acid. EPA can act as a competitive inhibitor of arachidonic acid conversion to these eicosanoids. Thus, these various fatty acids metabolism may interact with each other, and different effects may occur under different conditions of fatty acids intake among different populations. Further experimental and interventional studies under different conditions are needed to clarify these mechanisms and causal relationships.

Our study has several limitations. First, because the study is cross-sectional, we cannot infer causality from our results. Second, because intakes of oleic acid, linoleic acid, and ALA were strongly correlated with each other, independent relationships between each substance and CRP after adjustment for the other substances were not calculated in order to avoid multicollinearity. Third, although the validity of the DHQ was evaluated for total fat, SFA, MUFA, PUFA, EPA, and DHA,^15^^,^^16^ the validity for other specific fatty acids, including oleic acid, linoleic acid, and ALA, have not been fully established. Fourth, male participants may under-report their fat intake, as the total fat intake in men (20.8%E) was lower than that in previous reports in Japan. Although the reasons of relatively stronger relationships between CRP and specific fatty acids intake in women are not clear, one of the reasons might be lower reliability in fat intake in men.

In conclusion, this large-scale cross-sectional study in Japanese subjects suggested that intake of oleic acid, linoleic acid, and ALA would reduce serum CRP, especially when there is moderate intake of long-chain n-3 PUFAs (EPA+DHA). For the Japanese, the present level of seafood intake should be maintained, and, at the same time, substitution of plant-based PUFAs intake for SFAs is recommended. For Western populations, an increased intake of seafood may improve the anti-inflammatory effect of PUFAs.

## References

[1] Willerson JT, Ridker PM Inflammation as a cardiovascular risk factor. Circulation 2004;109[suppl II]:II2-II10. 15173056 10.1161/01.CIR.0000129535.04194.38

[2] Ridker PM, Buring JE, Cook NR, Rifai N C-reactive protein, the metabolic syndrome, and risk of incident cardiovascular events: an 8-year follow-up of 14 719 initially healthy American women. Circulation 2003;107:391-7. 12551861 10.1161/01.cir.0000055014.62083.05

[3] Ridker PM Role of inflammatory biomarkers in prediction of coronary heart disease. Lancet 2001;358:946-8. 11583743 10.1016/S0140-6736(01)06112-8

[4] Ridker PM, Hennekens CH, Buring JE, Rifai N C-reactive protein and other markers of inflammation in the prediction of cardiovascular disease in women. N Engl J Med 2000;342:836-42. 10733371 10.1056/NEJM200003233421202

[5] Visser M, Bouter LM, McQuillan GM, Wener MH, Harris TB Elevated C-reactive protein levels in overweight and obese adults. JAMA 1999;282:2131-5. 10591334 10.1001/jama.282.22.2131

[6] Yamada S, Gotoh T, Nakashima Y, Kayaba K, Ishikawa S, Nagao N, Distribution of serum C-reactive protein and its association with atherosclerotic risk factors in a Japanese population. Am J Epidemiol 2001;153:1183-90. 11415953 10.1093/aje/153.12.1183

[7] Zhao G, Etherton TD, Martin KR, West SG, Gillies PJ, Kris-Etherton PM Dietary *α* -linolenic acid reduces inflammatory and lipid cardiovascular risk factors in hypercholesterolemic men and women. J Nutr 2004;134:2991-7. 15514264 10.1093/jn/134.11.2991

[8] Railldis LS, Paschos G, Liakos GK, Velissaridou AH, Anastasiadis G, Zampelas A Dietary *α* -linolenic acid decreases C-reactive protein, serum amyloid A and interleukin-6 in dyslipidaemic patients. Atherosclerosis 2003;167:237-42. 12818406 10.1016/s0021-9150(02)00427-6

[9] Bemelmans WJ, Lefrandt JD, Feskens EJM, van Haelst PL, Broer J, Meyboom-de Jong B, Increased *α* -linolenic acid intake lowers C-reactive protein, but has no effect on markers of atherosclerosis. Eur J Clin Nutr 2004;58:1083-9. 15220952 10.1038/sj.ejcn.1601938

[10] Paschos GK, Rallidis LS, Liakos GK, Panagiotakos D, Anastasiadis G, Votteas V, Background diet influence the anti-inflammatory effects of *α* -linolenic acid in dyslipidaemic subjects. Brit J Nutr 2004;92:649-55. 15522134 10.1079/bjn20041230

[11] Basu A, Devaraj S, Jialal I Dietary factors that promote or retard inflammation. Arterioscler Thromb Vasc Biol 2006;26:995-1001. 16484595 10.1161/01.ATV.0000214295.86079.d1

[12] Mozaffarian D, Ascherio A, Hu FB, Stampfer MJ, Willet WC, Siscovick DS, Interplay between different polyunsaturated fatty acids and risk of coronary heart disease in men. Circulation 2005;111:157-64. 15630029 10.1161/01.CIR.0000152099.87287.83 PMC1201401

[13] Kris-Etherton PM, Taylor DS, Yu-Poth S, Huth P, Moriarty K, Fishell V, Polyunsaturated fatty acids in the food chain in the United States. Am J Clin Nutr 2000;71(suppl):179S-88S. 10617969 10.1093/ajcn/71.1.179S

[14] Sugano M, Hirahara F Polyunsaturated fatty acids in the food chain in Japan. Am J Clin Nutr 2000;71(suppl):189S-96S. 10617970 10.1093/ajcn/71.1.189S

[15] Sasaki S, Yanagibori R, Amano K Self-administered diet history questionnaire developed for health education: a relative validation of the test-version by comparison with 3-day diet record in women. J Epidemiol 1998;8:203-15. 9816812 10.2188/jea.8.203

[16] Sasaki S, Ushio F, Amano K, Morihara M, Todoriki T, Uehara Y, Serum biomarker-based validation of a self-administered diet history questionnaire for Japanese subject. J Nutr Sci Vitaminol 2000;46:285-96. 11227800 10.3177/jnsv.46.285

[17] Science and Technology Agency, Japan. Standard Tables of Food Composition in Japan. 5th ed. Tokyo: Printing Bureau of the Ministry of Finance, 2000 (in Japanese).

[18] Lopez-Garcia E, Schulze MB, Manson JE, Meigs JB, Albert CM, Rifai N, Consumption of (n-3) fatty acids is related to plasma biomarkers of inflammation and endothelial activation in women. J Nutr 2004;134:1806-11. 15226473 10.1093/jn/134.7.1806

[19] Geelen A, Brouwer IA, Schouten EG, Kluft C, Katen MB, Zock PL Intake of n-3 fatty acids from fish dose not lower serum concentrations of C-reactive protein in healthy subjects. Eur J Clin Nutr 2004;58:1440-2. 15100717 10.1038/sj.ejcn.1601986

[20] Chan DC, Watts GF, Barrett PH, Beilin LJ, Mori TA, Effect of atorvastain and fish oil on plasma high-sensitivity C-reactive protein concentrations in individuals with visceral obesity. Clin Chem 2002;48:877-83. 12029003

[21] Vega-Lopez S, Kaul N, Devaraj S, Cai RY, German B, Jialal I Supplementation with omega3 polyunsaturated fatty acids and all-rac alpha-tocopherol alone and in combination failed to exert an anti-inflammatory effect in human volunteers. Metabolism 2004;53:236-40. 14767877 10.1016/j.metabol.2003.09.012

[22] Esposito K, Marfella R, Ciotola M, Di Palo C, Giugliano G, D’Armiento M, Effects of a Mediterranean-Style diet on endothelial dysfunction and markers of vascular inflammation in the metabolic syndrome. JAMA 2004;292:1440-6. 15383514 10.1001/jama.292.12.1440

[23] Stamler J, Elliott P, Chan Q INTERMAP appendix tables. J Hum Hypertens 2003;17:665-775. 13679954 10.1038/sj.jhh.1001607 PMC6561117

[24] James MJ, Gibson RA, Cleland LG Dietary polyunsaturated fatty acids and inflammatory mediator production. Am J Clin Nutr 2000;71(suppl):343S-8S. 10617994 10.1093/ajcn/71.1.343s

[25] Calder PC Polyunsaturated fatty acids and inflammation. Biochem Soc Trans 2005;33:423-7. 15787620 10.1042/BST0330423

[26] Calder PC Dietary modification of inflammation with lipids. Proc Nutr Soc 2002;61:345-58. 12296294 10.1079/pns2002166

